# Entomopathogenic Nematodes in Soil Reduce Oviposition By the Leaf Herbivore *Ostrinia nubilalis*

**DOI:** 10.1111/pce.70795

**Published:** 2026-08-13

**Authors:** Paul Anton Himmighofen, Arletys Maria Verdecia‐Mogena, Sabine Kurz, Markus Frank, Gaetan Glauser, Pierre Mateo, Christelle Aurélie Maud Robert

**Affiliations:** ^1^ Institute of Plant Sciences University of Bern Bern Switzerland; ^2^ Institute of Applied Agriculture Nürtingen‐Geislingen University Nürtingen Germany; ^3^ Neuchâtel Platform of Analytical Chemistry University of Neuchâtel Neuchâtel Switzerland

## Abstract

Soil‐dwelling entomopathogenic nematodes induce systemic changes in maize that deter oviposition by *Ostrinia nubilalis*, revealing a plant‐mediated mechanism linking belowground natural enemies to aboveground herbivore host selection.

Tritrophic interactions between plants, herbivores, and herbivore enemies are critical in ecosystem functioning and diversity (Abdala‐Roberts et al. 2019). Increasing evidence indicates that plants can respond directly to organisms at the third trophic level, thereby altering multitrophic interactions (Kansman et al. 2025).

Entomopathogenic nematodes (EPNs) are soil‐dwelling parasitic roundworms widely used as biocontrol agents against insect herbivores (Dillman et al. 2012). Beyond directly infecting insects, EPNs can induce changes in plant metabolism (Verdecia‐Mogena et al. 2025; Kansman et al. 2025 and references therein). Such responses can increase plant resistance either through reduced herbivore performance (antibiosis) or through reduced host attractiveness and oviposition (antixenosis). For example, EPN exposure alters defence‐related enzymes, phytohormones, and primary metabolites in several plant species (Jagdale et al. 2009; Kamali et al. 2022), and has been associated with reduced herbivore performance and attraction (Helms et al. 2019; Kamali et al. 2022). However, the cues underlying EPN‐induced changes in herbivore behaviour remain poorly understood and can be mediated by both volatile and contact cues (Städler 2003). Additionally, field evidence remains scarce, and the agro‐ecological relevance of plant direct responses to EPNs in the field remains unclear.

In this study, we assessed whether EPN presence in soil influences maize interactions with the European corn borer (*Ostrinia nubilalis*). A field observation revealed a lower, albeit not significant, frequency of natural *O. nubilalis* infestation on EPN‐exposed maize plants (12.5% vs. 37.5% in controls; Figure 1A). A similar trend was observed in a second field assay (Supporting Figure S1). In controlled choice assays where females could land on the leaf surface, females laid more egg clutches on control than on EPN‐exposed plants (Figure 1B). These findings reinforce previous evidence about EPN‐induced antixenosis against leaf herbivores, such as in tomato leaf miners and Colorado potato beetles, although the involved cues mediating oviposition remained unclear (Helms et al. 2019; Kamali et al. 2022).

**Figure 1:**
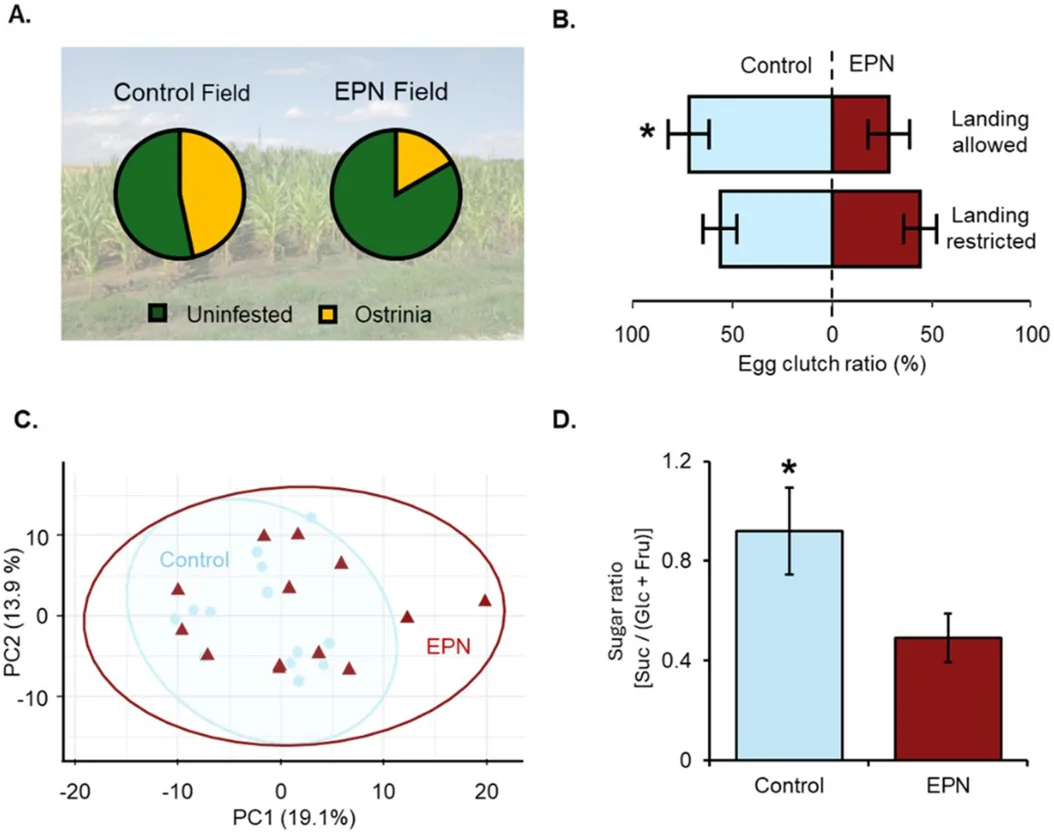
Entomopathogenic nematodes (EPNs) presence in the soil reduced *Ostrinia nubilalis* oviposition in maize plants. (A) Pie chart representing the rate of infestation by *O. nubilalis* on control and EPN exposed plants in the field (Control *n* = 15, EPN *n* = 12), in the background a view of the maize field used in this study (B) Ratio of egg clutches on maize plants without (“Landing allowed”, *n* = 10) or with a plastic mesh around (“Landing restricted”, *n* = 21). (C) PCA of the metabolomic profile of maize leaves (*n* = 13). (D) Ratio of leaf sucrose to the sum of glucose/fructose of control and EPN exposed plants under greenhouse conditions (*n* = 13). Plants were always inoculated with EPNs by infective juvenile addition into the soil close to the plant. Means ± standard errors are shown. Stars indicate significant differences (*p < 0.05). Detailed experimental procedures are provided in the Supporting Information S1.

Preventing *O. nubilalis* females to land on the plants, while allowing volatile cues to disperse, was sufficient to abolish the female preference for control over EPN‐exposed plants, highlighting that plant‐associated contact cues are required for selection (Figure 1B).

To assess whether belowground EPN presence triggers systemic molecular responses in maize leaves, we performed transcriptomic and metabolomic analyses 72 h after EPN inoculation. The presence of EPNs in soil was associated with treatment‐specific transcript detection patterns after 72 h, with 2144 transcripts detected exclusively in control plants and 2029 transcripts detected exclusively in EPN‐exposed plants (Supporting Figure S3). Representative differentially expressed genes (DEGs) included *LOC103635679* (log_2_FC = 22.72), *LOC100278145* (log_2_FC = 21.83), *LOC100037775* (log_2_FC = –22.56), and *LOC100274006* (log_2_FC = –22.05) (Supporting Table S1). Gene ontology enrichment analyses revealed significant overrepresentation of categories associated with immune and stress‐related processes, hypersensitive response, and immune response (Supporting Table S2). Pathway‐level analyses further highlighted enrichment in central metabolic processes, including pyruvate metabolism, propanoate metabolism, and fatty acid biosynthesis and metabolism (Supporting Figure S3). These enrichments suggest adjustments in core carbon flux and lipid metabolism. However, statistical analysis using DESeq. 2 identified only 45 significantly differentially expressed genes (FDR < 0.05) (Supporting Table S1). Moreover, independent qRT‑PCR analysis of 12 sugar transport and metabolism genes showed no significant differences between treatments. Untargeted metabolomic profiling did not reveal a global shift in leaf metabolite composition between control and EPN‐exposed plants (Figure 1C, PPLS‐DA: *p* = 0.86, classification error rate = 0.65). Principal component analysis showed substantial overlap between treatments (Supporting Figure S4). Hormone levels and volatile emission profiles similarly remained unchanged (Supporting Figure S4). This moderate change is consistent with a previous transcriptomic study on EPN‐induced changes in maize roots (Verdecia‐Mogena et al. 2025), and indicates that EPN presence induces coordinated yet subtle systemic adjustments rather than extensive metabolic remodelling.

Despite the overall stability of the metabolome, EPN exposure significantly altered soluble carbohydrate composition, decreasing the sucrose‐to‐(glucose+fructose) ratio from 0.92 in control plants to 0.49 in EPN‐treated plants (Figure 1D). Such shifts in carbohydrate balance may reflect changes in carbon allocation or source‐sink dynamics and could influence leaf surface chemistry. Because the oviposition effect depended on leaf contact, subtle modifications in primary metabolites may contribute to host acceptance decisions. Whether the decrease in the sucrose:glucose/fructose ratio directly mediates oviposition behaviour of *O. nubilalis* will require further investigation, for instance through surface‐choice assays in which defined sugar ratios are applied to neutral substrates before assessing female oviposition preference.

Interestingly, the female preference for control plants over EPN‐exposed plants did not enhance its progeny's performance (Supporting Figure S2), revealing a preference–performance mismatch. A similar behaviour in *O. nubilalis* was observed when selecting healthy over root‐infested maize plants (Hajdu et al. 2024). Alternatively, the females might select control over healthy plants to prevent their progeny from being infected by EPNs. No infection occurred during the feeding experiment, likely because EPNs do not crawl on the shoots to find a host. Since *O. nubilalis* larvae bore into the lower stem instead of soil for pupation, it is unclear at what stage EPNs may infect them. Finally, females may be responding to general plant cues triggered by the presence of nematodes. For instance, plants can react to EPNs with systemic responses that overlap with those induced by phytopathogenic nematodes (PPNs) (Manosalva et al. 2015), and such generalised defensive signalling may underlie altered herbivore behaviour.

In summary, this study demonstrates that belowground EPN presence can modulate aboveground herbivore oviposition through plant‐mediated, contact‐dependent mechanisms. Because EPN persistence was not monitored, one cannot distinguish between effects caused directly by EPN presence and those mediated indirectly through changes in the rhizosphere community. Resolving these mechanisms and their temporal dynamics represents an important avenue for future research. Nevertheless, these findings highlight an underappreciated pathway through which belowground natural enemies shape aboveground herbivore dynamics and suggest that EPNs may influence pest pressure not only through direct parasitism but also by altering plant attractiveness within tritrophic networks.

## Conflicts of Interest

The authors declare no conflicts of interest.

## Supporting information


**Supporting Information S1:** Materials and Methods.

**Figure S1**: EPN presence reduced infestation by *Ostrinia nubilalis* in an independent field assay. **Figure S2**: Number of eggs per clutch, larval performance, and survival of *Ostrinia nubilalis* on control and EPN‐exposed plants. **Figure S3**: Transcriptomic response of maize plants to EPN‐exposure. **Figure S4**: Metabolomic response of maize plants to EPN‐exposure.

**Table S1**: Differentially expressed genes (DEGs) in maize leaves after EPN‐exposure. **Table S2**: Gene Ontology (GO) terms enrichment analysis in maize leaves after EPN exposure.

## Data Availability

The transcriptomic data will be deposited on the NCBI repository (https://www.ncbi.nlm.nih.gov/sra). All raw data is available upon request.
