# Supplementary material for: Entomopathogenic Nematodes in Soil Reduce Oviposition By the Leaf Herbivore Ostrinia nubilalis
Source: Plant Cell Environ. 2026 Aug 13;49(11):9265–7. doi: 10.1111/pce.70795 (PMC13646685; doi:10.1111/pce.70795)
Supplement: Supplementary file 1 [file PCE-49-9265-s001.docx]

## Brief communication

# **Entomopathogenic nematodes in soil reduce oviposition by the leaf herbivore *Ostrinia nubilalis***

P. A. Himmighofen^1,‡^, A. M. Verdecia-Mogena^1,‡^, S. Kurz^2^, M. Frank^2^, G. Glauser^3^, P. Mateo^1^, C. A.M. Robert^1^*

^1^Institute of Plant Sciences, University of Bern, Altenbergrain 21, 3013 Bern, Switzerland; ^2^Institute of Applied Agriculture, Nürtingen-Geislingen University, Nürtingen, Germany. ^3^ Neuchâtel Platform of Analytical Chemistry, University of Neuchâtel, Avenue de Bellevaux 51, 2000 Neuchâtel, Switzerland

^‡^ Equal first co-authorship; * Corresponding author

Supplementary Tables

# Supplementary Table S1.

**Supplementary Table S1**: Gene Ontology (GO) terms enrichment analyses of molecular function (MF), biological processes (BP), and cellular components (CC) in maize shoots after 72 h exposure to entomopathogenic nematodes (EPNs). EPN exposure was achieved by adding 2’000 infective juveniles of *Heterorhabditis bacteriophora* nematodes for 72 h in the soil (n = 5).

| **Source** | **Term name** | **Term id** | **Adjusted p_value** | **Term size** | **Intersection size** |
| --- | --- | --- | --- | --- | --- |
| GO:MF | molecular_function | GO:0003674 | 0.001 | 24540 | 36 |
| GO:MF | ATP binding | GO:0005524 | 0.027 | 3786 | 10 |
| GO:MF | carbohydrate derivative binding | GO:0097367 | 0.027 | 4474 | 11 |
| GO:MF | gamma-glutamylaminecyclotransferase activity | GO:0061929 | 0.027 | 2 | 1 |
| GO:MF | 4-amino-4-deoxychorismate synthase activity | GO:0046820 | 0.027 | 1 | 1 |
| GO:MF | adenyl ribonucleotide binding | GO:0032559 | 0.027 | 3903 | 11 |
| GO:MF | ribonucleotide binding | GO:0032553 | 0.027 | 4438 | 11 |
| GO:MF | adenyl nucleotide binding | GO:0030554 | 0.027 | 4071 | 11 |
| GO:MF | DNA helicase activity | GO:0003678 | 0.027 | 241 | 3 |
| GO:MF | binding | GO:0005488 | 0.027 | 16538 | 26 |
| GO:MF | purine ribonucleotide binding | GO:0032555 | 0.027 | 4393 | 11 |
| GO:MF | nucleoside phosphate binding | GO:1901265 | 0.028 | 4865 | 11 |
| GO:MF | heterocyclic compound binding | GO:1901363 | 0.028 | 5056 | 11 |
| GO:MF | ATP-dependent activity | GO:0140657 | 0.028 | 1529 | 6 |
| GO:MF | nucleotide binding | GO:0000166 | 0.028 | 4857 | 11 |
| GO:MF | ATP-dependent activity, acting on DNA | GO:0008094 | 0.028 | 373 | 3 |
| GO:MF | catalytic activity | GO:0003824 | 0.028 | 14404 | 22 |
| GO:MF | nucleic acid binding | GO:0003676 | 0.028 | 5792 | 12 |
| GO:MF | protein binding | GO:0005515 | 0.028 | 5352 | 12 |
| GO:MF | purine nucleotide binding | GO:0017076 | 0.028 | 4563 | 11 |
| GO:MF | phosphatidylinositol N-acetylglucosaminyltransferase activity | GO:0017176 | 0.028 | 5 | 1 |
| GO:MF | NEDD8 activating enzyme activity | GO:0019781 | 0.028 | 5 | 1 |
| GO:MF | purine ribonucleoside triphosphate binding | GO:0035639 | 0.028 | 4255 | 10 |
| GO:MF | isomerase activity | GO:0016853 | 0.028 | 1078 | 5 |
| GO:MF | macromolecular conformation isomerase activity | GO:0120543 | 0.028 | 652 | 4 |
| GO:MF | anion binding | GO:0043168 | 0.028 | 5019 | 11 |
| GO:MF | ditrans,polycis-polyprenyl diphosphate synthase [(2E,6E)-farnesyl diphosphate specific] activity | GO:0045547 | 0.028 | 4 | 1 |
| GO:MF | four-way junction DNA binding | GO:0000400 | 0.029 | 6 | 1 |
| GO:MF | four-way junction helicase activity | GO:0009378 | 0.033 | 7 | 1 |
| GO:MF | acetyl-CoA carboxylase activity | GO:0003989 | 0.033 | 8 | 1 |
| GO:MF | catalytic activity, acting on a nucleic acid | GO:0140640 | 0.033 | 1307 | 5 |
| GO:MF | nuclear export signal receptor activity | GO:0005049 | 0.033 | 8 | 1 |
| GO:MF | ion binding | GO:0043167 | 0.033 | 8536 | 15 |
| GO:MF | CoA carboxylase activity | GO:0016421 | 0.035 | 9 | 1 |
| GO:MF | ligase activity, forming carbon-carbon bonds | GO:0016885 | 0.035 | 9 | 1 |
| GO:MF | small molecule binding | GO:0036094 | 0.038 | 8756 | 15 |
| GO:MF | helicase activity | GO:0004386 | 0.041 | 484 | 3 |
| GO:MF | nucleic acid conformation isomerase activity | GO:0120545 | 0.045 | 508 | 3 |
| GO:MF | cytoskeletal protein binding | GO:0008092 | 0.046 | 517 | 3 |
| GO:BP | cellular process | GO:0009987 | 0.000 | 20416 | 34 |
| GO:BP | biological_process | GO:0008150 | 0.000 | 21625 | 35 |
| GO:BP | cellular response to stress | GO:0033554 | 0.007 | 1090 | 7 |
| GO:BP | plant-type hypersensitive response | GO:0009626 | 0.013 | 21 | 2 |
| GO:BP | symbiont-induced defense-related programmed cell death | GO:0034050 | 0.013 | 21 | 2 |
| GO:BP | programmed cell death | GO:0012501 | 0.029 | 52 | 2 |
| GO:BP | macromolecule metabolic process | GO:0043170 | 0.029 | 11370 | 20 |
| GO:BP | innate immune response | GO:0045087 | 0.029 | 53 | 2 |
| GO:BP | nucleobase-containing compound metabolic process | GO:0006139 | 0.029 | 6167 | 14 |
| GO:BP | response to stress | GO:0006950 | 0.029 | 2309 | 8 |
| GO:BP | regulation of phosphate transport | GO:0010966 | 0.029 | 1 | 1 |
| GO:BP | organelle organization | GO:0006996 | 0.029 | 1649 | 7 |
| GO:BP | cell death | GO:0008219 | 0.029 | 54 | 2 |
| GO:BP | nucleic acid metabolic process | GO:0090304 | 0.029 | 5515 | 13 |
| GO:BP | immune response | GO:0006955 | 0.029 | 55 | 2 |
| GO:BP | immune system process | GO:0002376 | 0.029 | 55 | 2 |
| GO:BP | dolichol biosynthetic process | GO:0019408 | 0.037 | 2 | 1 |
| GO:BP | para-aminobenzoic acid metabolic process | GO:0046482 | 0.037 | 2 | 1 |
| GO:BP | 4-aminobenzoate biosynthetic process | GO:0008153 | 0.037 | 2 | 1 |
| GO:BP | primary metabolic process | GO:0044238 | 0.044 | 13856 | 22 |
| GO:BP | defense response to symbiont | GO:0140546 | 0.044 | 77 | 2 |
| GO:BP | biosynthetic process | GO:0009058 | 0.045 | 8513 | 16 |
| GO:CC | cellular_component | GO:0005575 | 0.000 | 20598 | 36 |
| GO:CC | organelle | GO:0043226 | 0.000 | 12525 | 27 |
| GO:CC | intracellular anatomical structure | GO:0005622 | 0.000 | 14504 | 29 |
| GO:CC | intracellular organelle | GO:0043229 | 0.000 | 12512 | 27 |
| GO:CC | cellular anatomical structure | GO:0110165 | 0.000 | 20322 | 34 |
| GO:CC | membrane-bounded organelle | GO:0043227 | 0.000 | 11276 | 24 |
| GO:CC | intracellular membrane-bounded organelle | GO:0043231 | 0.000 | 11250 | 24 |
| GO:CC | transferase complex | GO:1990234 | 0.002 | 849 | 6 |
| GO:CC | protein-containing complex | GO:0032991 | 0.004 | 4307 | 12 |
| GO:CC | cytoplasm | GO:0005737 | 0.004 | 8776 | 18 |
| GO:CC | dehydrodolichyl diphosphate synthase complex | GO:1904423 | 0.008 | 1 | 1 |
| GO:CC | polyprenyl diphosphate synthase complex | GO:0032476 | 0.008 | 1 | 1 |
| GO:CC | TRAMP complex | GO:0031499 | 0.014 | 2 | 1 |
| GO:CC | catalytic complex | GO:1902494 | 0.016 | 1479 | 6 |
| GO:CC | endoplasmic reticulum | GO:0005783 | 0.016 | 1019 | 5 |
| GO:CC | RNA N6-methyladenosine methyltransferase complex | GO:0036396 | 0.017 | 3 | 1 |
| GO:CC | nucleus | GO:0005634 | 0.020 | 6329 | 13 |
| GO:CC | cellulose synthase complex | GO:0010330 | 0.029 | 6 | 1 |
| GO:CC | glycosylphosphatidylinositol-N-acetylglucosaminyltransferase (GPI-GnT) complex | GO:0000506 | 0.029 | 6 | 1 |
| GO:CC | endoplasmic reticulum subcompartment | GO:0098827 | 0.034 | 469 | 3 |
| GO:CC | microbody | GO:0042579 | 0.034 | 139 | 2 |
| GO:CC | nuclear outer membrane-endoplasmic reticulum membrane network | GO:0042175 | 0.034 | 468 | 3 |
| GO:CC | mitochondrial DNA-directed RNA polymerase complex | GO:0034245 | 0.034 | 9 | 1 |
| GO:CC | peroxisome | GO:0005777 | 0.034 | 139 | 2 |
| GO:CC | organelle membrane | GO:0031090 | 0.034 | 1980 | 6 |
| GO:CC | peroxisomal importomer complex | GO:1990429 | 0.034 | 9 | 1 |
| GO:CC | endoplasmic reticulum membrane | GO:0005789 | 0.034 | 466 | 3 |

# Supplementary Table S2.

**Supplementary Table S2**: Differentially expressed genes (DEGs) in maize shoots after 72 h exposure to entomopathogenic nematodes (EPNs). EPN exposure was achieved by adding 2’000 infective juveniles of *Heterorhabditis bacteriophora* nematodes for 72 h in the soil (n = 5).

| **Symbol** | **log2 fold change (EPNs/Control)** | **Q value** |
| --- | --- | --- |
| LOC100283559 | 0.200212 | 0.022471 |
| LOC100502274 | 7.238945 | 0.015468 |
| LOC103629626 | 0.462725 | 0.015468 |
| LOC100278145 | 20.67334 | 2.25E-08 |
| LOC100278145 | 21.83331 | 3.01E-09 |
| LOC103629717 | -5.97963 | 6.58E-05 |
| LOC103630717 | -0.68791 | 0.001189 |
| LOC100276916 | -7.10687 | 1.74E-08 |
| LOC100284792 | 21.69724 | 3.01E-09 |
| LOC100285165 | 6.261829 | 0.001189 |
| LOC541775 | -7.5206 | 0.001155 |
| LOC103631855 | -0.3884 | 0.000743 |
| LOC103633205 | -7.49358 | 0.001411 |
| LOC100283640 | 7.315467 | 0.004431 |
| LOC100037775 | -22.5561 | 1.18E-09 |
| LOC103642154 | 8.569844 | 0.009428 |
| LOC103643327 | -8.58612 | 0.01514 |
| LOC100276550 | 7.621619 | 3.01E-09 |
| LOC100279211 | -21.7129 | 3.01E-09 |
| LOC100279983 | -5.30202 | 9.51E-12 |
| LOC103651688 | -6.45442 | 8.7E-05 |
| LOC103654570 | -21.7443 | 3.01E-09 |
| LOC103629593 | 21.30543 | 6.29E-09 |
| LOC100382790 | -6.59364 | 0.034229 |
| LOC103635679 | 22.71626 | 1.04E-09 |
| LOC103637423 | -6.88786 | 0.002773 |
| LOC103638902 | 6.762323 | 0.022912 |
| LOC100273625 | -21.3931 | 5.6E-09 |
| LOC100384813 | -21.7146 | 3.01E-09 |
| LOC103641968 | -8.96692 | 5.07E-14 |
| LOC100037775 | 8.225457 | 6.8E-05 |
| LOC103626416 | -1.13336 | 0.021516 |
| LOC100382683 | -2.15154 | 0.003942 |
| LOC100383420 | 20.01174 | 9.12E-08 |
| LOC103629927 | 20.7995 | 1.76E-08 |
| LOC103630113 | 20.57277 | 2.71E-08 |
| LOC103636243 | 0.702868 | 0.004583 |
| LOC100193888 | -21.5692 | 3.91E-09 |
| LOC103639007 | -7.63343 | 0.001138 |
| LOC103641468 | -5.17523 | 0.007479 |
| LOC103643254 | -6.50134 | 4.48E-05 |
| LOC103643456 | -21.7118 | 3.01E-09 |
| LOC100279951 | 20.88638 | 1.6E-08 |
| LOC103646242 | -21.7302 | 3.01E-09 |
| LOC100274006 | -22.054 | 3.01E-09 |
