# Supplementary material for: Entomopathogenic Nematodes in Soil Reduce Oviposition By the Leaf Herbivore Ostrinia nubilalis
Source: Plant Cell Environ. 2026 Aug 13;49(11):9265–7. doi: 10.1111/pce.70795 (PMC13646685; doi:10.1111/pce.70795)
Supplement: Supplementary file 2 [file PCE-49-9265-s002.docx]

## Brief communication

# **Entomopathogenic nematodes in soil reduce oviposition by the leaf herbivore *Ostrinia nubilalis***

P. A. Himmighofen^1,‡^, A. M. Verdecia-Mogena^1,‡^, S. Kurz^2^, M. Frank^2^, G. Glauser^3^, P. Mateo^1^, C. A.M. Robert^1^*

^1^Institute of Plant Sciences, University of Bern, Altenbergrain 21, 3013 Bern, Switzerland; ^2^Institute of Applied Agriculture, Nürtingen-Geislingen University, Nürtingen, Germany. ^3^ Neuchâtel Platform of Analytical Chemistry, University of Neuchâtel, Avenue de Bellevaux 51, 2000 Neuchâtel, Switzerland

^‡^ Equal first co-authorship; * Corresponding author

Supplementary Information S1. Materials and Methods

Biological resources

Maize seeds (*Zea mays* L.) of the variety B73 were kindly provided by Maize GDB ([www.maizegdb.org](https://www.maizegdb.org/)) and bred by Delley DSP (Delley Seeds and Plants Ltd, Delley, Switzerland). Maize seeds of the variety Amaveritas were bought from Agromais (Agromais GmbH, Everswinkel, Germany). Maize seeds of the variety Akku were bought from Delley DSP (Delley Seeds and Plants Ltd, Delley, Switzerland). Plants grown in greenhouse conditions were sown in cylindrical 100 mL plastic pots (11 cm height x 4 cm diameter, Semadeni, Ostermundigen, Switzerland), 1 L or 10 L plastic pots (Lambrecht-Verpackungen GmbH, Göttingen, Germany) depending on the duration of the experiment. All plants were grown in soil (Selmaterra Schweizer Schwererde torfreduziert, Bigler Samen AG, Thun, Switzerland) and kept in a greenhouse at 23 +/- 2°C, approx. 60 % humidity and a day:night cycle of 16:8 hours. Fertilizer (Plantaktiv® Typ K, Hauert HBG Dünger AG, Grossaffoltern, Switzerland) was added once a week (0.1-0.3 % w/v) following the manufacturer instructions.

Eggs of *Ostrinia nubilalis* were bought from BTL Bio-Test Labor GmbH Sagerheide (Groß Lüsewitz, Germany) and reared on general purpose lepidoptera diet (Frontier Agricultural Sciences, DE, USA) and young maize leaves (var. Akku).

Entomopathogenic nematodes (*Heterorhabditis bacteriophora* Poinar, EN01) were bought from Andermatt Biocontrol (Grossdietwil, Switzerland) and reared *in vivo* in greater wax moth larvae (*Galleria melonella* Lepidoptera: Pyralidae, Fischereibedarf Wenger, Bern, Switzerland). For the field trial, *H. bacteriophora* (EN01) infective juveniles (IJs) were kindly provided by e-nema GmbH (Schwentinental, Germany) and used directly without prior rearing in *G. melonella*. IJ EPNs were used for all experiments.

Natural herbivore infestation in the field

A field study was conducted in 2021 at Tachenhausen at the agricultural facilities of the Nürtingen-Geislingen University, Germany (48°39'08.9"N 9°23'20.7"E). In May, Amaveritas maize seeds were grown as buffer plants at 8 plants per m^2^ with 75 cm row spacing. Single B73 maize seeds were grown every third row of the hybrid variety at a 1.5 m distance between plants, to ensure that EPNs cannot travel between B73 plants. The treatment and control plants were randomly assigned among B73 plants distributed across the field. The field was then treated with the herbicides Stomp and Spectrum (BASF, Ludwigshafen am Rhein, Germany) at a concentration of 2.8 and 1.4 L/hectare, respectively. One month later, EPN-treated plants were inoculated with 10’000 live EPN IJs (n = 80). The inoculation was performed by adding 50 mL of tap water containing IJs into a 5 cm deep hole, dug 5 cm away from the stem. Control plants underwent the same procedure but received 50 mL of tap water without IJs. EPN inoculation was repeated monthly over three months. Plant height was measured bimonthly. Chlorophyll content was measured by proxy with a SPAD-502 (Konica Minolta Sensing, Nieuwegein, Netherlands) at bottom, middle and top of a medium sized leaf sheath. The presence of *O. nubilalis* larvae and associated damage were recorded on a subset of plants (Control n = 15, EPN n = 12) in August at the flowering stage. No specific permits were required to conduct the field experiment, as it did not involve genetically modified organisms or pesticide registration trials.

Insect performance

Pre-germinated seedlings were transferred into 1 L pots (Lambrecht-Verpackungen GmbH, Göttingen, Germany) with soil Selmaterra Schweizer Schwererde torfreduziert, Bigler Samen AG, Thun, Switzerland). After five to six weeks of growth under greenhouse conditions, 2’000 EPN IJs were applied in 10 mL tap water by creating a 2–3 cm deep hole in the soil approximately 4 cm away from the stem to minimize root damage (n = 13). Control plants received water only (n = 13). After 24 h, three pre-weighed third-instar *O. nubilalis* larvae were placed into the leaf whorl of each plant. The plants were then covered with air- and humidity-permeable fleece (Pflanzen-Winterschutz-Vlies, 17 g/m², Tebo AG, Windisch, Switzerland) to prevent larval escape. Seven days later, the larvae were collected and weighed. Leaf tissue was harvested and immediately frozen in liquid nitrogen for subsequent metabolomic, phytohormone, sugar, and amino acid analyses.

Insect oviposition preference

Oviposition preference was assessed in two independent experiments. In a first experiment, two ‑week ‑old maize plants were opposite each other within a dome-shaped cage (BugDorm 2S120, W60 x D60 x H60 cm, 680 µm aperture, Megaview Science Co., Ltd, Taiwan). One of the plants was treated with 2’000 EPNs in 10 mL of tap water applied at 3 cm from the stem, while the second plant received 10 mL of tap water without IJs. A dish containing cotton soaked in honey water (concentration 3 %) was placed between the plants to provide nutrients for the *O. nubilalis* moths. Two hours later, three female moths were released into each cage and allowed to oviposit for 72 h. The number of egg clutches and eggs per clutch were then recorded for each plant (n = 5). Egg clutches were counted when found on the plant itself or the pot. The experiment was repeated two times to ensure sufficient replicate number (n = 10).

In a second experiment, the plants offered to *O. nubilalis* females were covered with perforated plastic bags (oven bags, Tangan N° 34, Migros, Zurich, Switzerland) to prevent *O. nubilalis* females from landing on the plant and using contact cues. Egg clutches placed on the bags or the pot were counted. The experiment was repeated four times to ensure sufficient replicate number (n=30). Cages in which females did not deposit any eggs on a plant or a pot were excluded from the preference analysis because no host-choice decision could be inferred (n=9).

Transcriptomic analysis

IJ suspensions of about 500 IJs/ml were prepared in tap water and were added into two 2 cm deep holes in the soil (2’000 EPNs per pot). Same volumes of water were added to control plants. After 72 h maize shoots were collected and snap-frozen in liquid nitrogen. Samples were grinded to a fine powder in liquid nitrogen using a mortar and pestle. Total RNA was extracted from 100 ± 2 mg ground maize leaves using a RNeasy Plant Mini Kit (QIAGEN Naamloze Vennootschap (N. V.), Venlo, The Netherlands) according to the manufacturer’s instructions (n = 5). The RNA integrity was evaluated using an Agilent 2100 Bioanalyzer (Agilent Technologies Inc, Santa Clara, California, USA). Samples with an RNA Integrity Number (RIN) ≥ 7 were used for subsequent analyses. The libraries were sequenced in a DNBSEQTM platform by Beijing Genomics Institute (BGI) (BGI Hong Kong Company Limited, Hong Kong, China) which generated 100 bp paired-end reads and 30 M reads per sample. Low quality reads, reads with adaptor sequences, and reads with high levels of N base were removed from the raw data with the filtering software SOAPnuke (Version v1.5.2). Hierarchical Indexing for Spliced Alignment of Transcripts (HISAT; Version v2.0.4) was used to map RNA-seq reads (Reference Genome version: GCF_902167145.1_Zm-B73-REFERENCE-NAM-5.0) and Bowtie2 tool was used to align the clean reads to the reference genes.

Gene-level read counts were normalized using the DESeq2 size‑factor method. Differential expression analysis was performed using the DESeq2 negative binomial model implemented in the Dr. Tom analysis system (BGI). Genes with a false discovery rate (FDR) ≤ 0.05 (Benjamini–Hochberg correction) were considered differentially expressed. No fold‑change threshold was applied. The resulting differentially expressed genes (DEGs) were used for downstream enrichment analyses.

Gene Ontology (GO) functional significant enrichment analysis gave the GO functional entries (terms) that are significantly enriched in candidate genes compared to the full genetic background of the species. For this analysis, first they mapped all candidate genes to each entry in the Gene Ontology database ([www.geneontology.org](http://www.geneontology.org/)), calculates the number of genes per entry, and then applies a hypergeometric test to find the GO function that is significantly enriched in candidate genes compared to all background genes of the specie. Then, they calculated the P value using stats package (Version 4.4.0) of R. The P value was corrected by multiple testing with Bioconductor package (Version 4.3) as Q value. Finally, Q value (corrected *p*‑value ≤ 0.05 was used as the threshold), and the GO term that satisfied this condition was defined as the GO term that was significantly enriched in candidate genes. GO functional enrichment profiling plots were generated on g:Profiler version *e113_eg59_p19_f6a03c19*, database updated on 23/05/2025 ([gprofiler/gost](https://biit.cs.ut.ee/gprofiler/gost)).

Kyoto Encyclopedia of Genes and Genomes (KEGG) analysis was done with the same methodology as the GO functional enrichment analysis, as described above for GO enrichment. Pathways with a final Q value ≤ 0.05 were defined as those significantly enriched in DEGs, and significant enrichment by pathway identified the most important biochemical metabolic and signalling pathways involved in candidate genes. The DEGs were further analysed and KEGG enrichment pathways analyses using Dr. Tom, a BGI customized data mining system ([biosys.bgi.com](https://biosys.bgi.com/%23/report/login)). Network analyses were conducted on ShinyGo 0.77, a graphical gene-set enrichment online tool (Ge et al., 2019).

Metabolomic analyses

Metabolomic profiles of leaf samples from the insect performance assay (n = 13) were analysed with an Acquity Ultra-High Performance Liquid Chromatography (UHPLC) system coupled to a G2-XS quadrupole Time-Of-Flight Mass Spectrometer (qTOF-MS) equipped with an electrospray source (Waters, Milford, MA, USA) as previously described (Sun et al., 2022). Briefly, the chromatography was performed using a Ethylene Bridged Hybrid (BEH) C18 column (100 × 2.1 mm i.d., 1.7 μm particle size) using the following elution profile: 2–50% B over 4 min, 50–100% B over 2 min, 100% B for 2 min, followed by re-equilibration with 2% B for 2 min, where A = 0.1% formic acid (FA) (Fisher Chemical, Waltham, MA, USA) in ultra-pure MilliQ water and B = 0.1% FA (Fisher Chemical) in acetonitrile (ACN) (Biosolve Chimie, Dieuze, France). The flow rate and temperature of the column were maintained at 0.4 mL.min^−1^ and 40 °C, respectively. The qTOF-MS operated in positive mode. The electrospray capillary and sampling cone voltages were set to 2 kV and 20 V, respectively. The source temperature was kept at 140 °C and the desolvation gas temperature at 400 °C. The desolvation and cone gas flows were 1000 L.hr-1 and 100 L.hr-1, respectively. Centroided data were acquired over a range of 50-1’200 m/z in MSE mode, using alternating scans of 0.1 s at low collision energy of 6 eV and 0.1 s at high collision energy ramped from 10 to 30 eV. The resulting chromatograms were aligned, deconvoluted and normalized with Progenesis QI v.2.4 (Waters, Milford, MA, USA).

Phytohormone analysis

The phytohormones in leaf tissue from the insect performance assay (n = 13) were extracted and analysed according to an adapted protocol described in (Glauser et al., 2014). Briefly, fresh frozen leaf tissue was ground to a fine powder in liquid nitrogen. Then, 50 mg of powder were mixed with 500 μL of extraction solvent (10 % Methanol [MeOH, Fisher Chemical] + 0.2 % FA [Fisher Chemical, Waltham, MA, USA]) and 10 μL of internal standard solution (100 ng/mL d_5_-JA, d_6_-ABA, d_6_-Salicylic acid [SA], d_5_-Indole acetic acid [IAA] [CDN Isotopes Inc., Point-Claire, Canada] and ^13^C_6_-JA-Ile [provided by Gaétan Glauser, Neuchâtel]). The mixture was extracted for 3 min at 30 Hz in a mixer mill (Retsch, Haan, Deutschland). After centrifugation at 15’000 *g*, the supernatant was removed and mixed with 500 μL of MilliQ water. This mixture was loaded onto an Oasis MAX solid phase extraction (SPE) column (30 mg, 1cc, Waters, Milford, MA, USA) after equilibration of the column using MeOH and 95:5 H_2_O:MeOH + 0.1 % FA. Then, columns were washed with a 95:5 H_2_O:NH_3_ (25 %, Fluka Chemie AG, Buchs, Switzerland) solution and MeOH + 0.2 % NH_3_ (25 %). Afterwards, the phytohormones were eluted from the columns with a 98:2 MeOH:FA solution. Finally, the eluent was evaporated in a CentriVap (Labconco Corp, MO, USA) and phytohormones were redissolved in 200 μL of 35 % MeOH. Samples were analysed using a UHPLC-MS/MS targeted approach (Glauser et al., 2014). The analysis was performed on an Acquity UHPLC system equipped with a BEH C18 column (50 x 2.1 mm, 1.7 µm) and coupled to a QTRAP 6500+ (AB Sciex LLC, Framingham, MA, USA) operating in both negative and positive modes.

Sugar analysis

Soluble sugars from leaf tissue were quantified using a Sucrose/D-Glucose/D-Fructose assay kit (Megazyme, Bray, Ireland) according to the manufacturer instructions (n = 13 per treatment). Sugars were extracted by mixing 50 mg of finely ground frozen tissue powder with 250 μL of 80 % Ethanol (EtOH, Fisher Chemical). This mixture was heated to 78°C while shaking at 800 rpm for 15 min. Afterwards, extracts were centrifuged for 10 min at 15’000 g and supernatant removed. This step was repeated twice more with 50 % EtOH and supernatants pooled for the assay. The preparation of the samples was done in transparent, flat-bottom 96-well plates (Dynatec laboratories, El Paso, TX, USA) and the measurements were performed on a Tecan Infinite M200PRO plate reader (Tecan Group Ltd., Männedorf, Switzerland).

Amino acid analysis

Free amino acids were quantified using the AccQ-Tag Ultra Derivatization Kit (Waters, Milford, USA) according to the manufacturer instructions (n = 10 per treatment). Amino acids were extracted by mixing 100 mg of tissue powder with 1 mL of extraction buffer (50 % EtOH + 0.1 % FA). The mixture was vortexed and centrifuged at 15’000 x *g* for 10 minutes. The supernatant was removed and evaporated to dryness in vacuum under 45 °C in a CentriVap (Labconco Corporation, Kansas City, USA). Free amino acids were redissolved in 400 μL of sterile MilliQ water obtained from a Barnstead™ Nanopure™ (Thermo Fisher Scientific Inc, Waltham, USA). Derivatized samples were analysed with an Acquity UHPLC system coupled to a Quadrupole Dalton-based Mass Spectrometer (QDa-MS) equipped with an electrospray source and an UV/Vis-Detector (Waters, Milford, MA, USA). The chromatography was operated with a BEH C18 column (100 × 2.1 mm i.d., 1.7 μm particle size, REF provider) using the following elution gradients: 0.1 % B for 0.54 min, 0.1-9.1 % B over 5.2 min, 9.1-21.2 % B over 2 min, 21.2-59.6 % B for 0.3 min, followed by flushing the column with 90 % B for 0.6 min and re-equilibration at 0.1 % B for 0.89 min (A = H20:ACN 99:1 + 0.1 % FA, B = ACN + 0.1 % FA). The flow rate and temperature of the column was kept constant at 0.7 mL/min) and 55 °C. The QDa-MS was operated in positive, single ion recording mode. The electrospray voltage was kept at 0.8 kV, while the cone voltage was adjusted according to the measured ions. The source and probe temperature were kept at 120 °C and 600 °C, respectively. The sampling frequency was set to 8 Hz. The chromatographic data obtained was processed in QuanLynx v.4.1 (Waters, Milford, USA) and amino acid concentrations were quantified using a mixture of 17 amino acids as external standards: Alanine, Arginine, Aspartic acid, Cystine, Glutamic acid, Glycine, Histidine, Isoleucine, Leucine, Lysine, Methionine, Phenylalanine, Proline, Serine, Threonine, Tyrosine, Valine (Waters, Milford, Massachusetts, USA, p/n: WAT088122).

Statistical analysis

Data obtained was analysed using R ([www.r-project.org](https://www.r-project.org/), version 4.2.2). AI supported the processes of codes improvement for R scripts. Plant growth and targeted analysis data were tested for normality and homogeneity of variance. Height measurements over time were analysed using linear regression models. Field height data were analysed using Type III ANOVAs, as the design was unbalanced with less plants available at each time point due to destructive harvests and damage by a hailstorm in June 2021. Infestation of *O. nubilalis* in the field was analysed with a Fisher’s exact test, and oviposition preference with a one sample t-test on eggs ratios on each plant compared to the null hypothesis of 1 (equal oviposition). Two-sample tests were performed using Welch’s test for parametric data and the Wilcoxon-rank-sum test for non-parametric data.

Metabolomic data were analysed using principal component analysis (PCA) and powered partial least squares – distribution analysis (PPLS-DA). For all analyses, background noise from samples was removed by discarding peaks present in blank measurements at a signal:noise ratio of 5:1. Features before 0.7 min retention time were excluded. Retention time of measured features were rounded to 1/100 minutes and the most abundant ion feature at each retention time was selected for analysis. Data was log transformed and scaled using the default scale() function to account for high differences in relative ion abundances. False Discovery Rate was tested using Benjamini-Hochberg correction. Cross validation was performed using the MVA.test and MVA.cmv functions in the package RVAideMemoire (Version 0.9-81-2). To create and visualize PCAs, the packages FactoMineR (Version 2.4), factoextra (Version 1.0.7) and ggplot2 (Version 3.3.6) were used. PPLS-DAs were calculated using the MVA.test and MVA.cmv function in the package RVAideMemoire (Version 0.9-81-2).
