# Supplementary material for: Entomopathogenic Nematodes in Soil Reduce Oviposition By the Leaf Herbivore Ostrinia nubilalis
Source: Plant Cell Environ. 2026 Aug 13;49(11):9265–7. doi: 10.1111/pce.70795 (PMC13646685; doi:10.1111/pce.70795)
Supplement: Supplementary file 3 [file PCE-49-9265-s003.docx]

## Brief communication

# **Entomopathogenic nematodes in soil reduce oviposition by the leaf herbivore *Ostrinia nubilalis***

P. A. Himmighofen^1,‡^, A. M. Verdecia-Mogena^1,‡^, S. Kurz^2^, M. Frank^2^, G. Glauser^3^, P. Mateo^1^, C. A.M. Robert^1^*

^1^Institute of Plant Sciences, University of Bern, Altenbergrain 21, 3013 Bern, Switzerland; ^2^Institute of Applied Agriculture, Nürtingen-Geislingen University, Nürtingen, Germany. ^3^ Neuchâtel Platform of Analytical Chemistry, University of Neuchâtel, Avenue de Bellevaux 51, 2000 Neuchâtel, Switzerland

^‡^ Equal first co-authorship; * Corresponding author

# Supplementary Figure S1.

**Supplementary Figure S1: EPN presence reduced infestation by *Ostrinia nubilalis* in an independent field assay.** Plants were inoculated with *Heterorhabditis bacteriophora* infective juveniles once per month in the season (Multiple application) or with a mock (Control). Presence of *O. nubilalis* damage or larvae was assessed in a subset of control (n = 7) and EPN-treated (n = 4) plants.

# Supplementary Figure S2.

**Supplementary Figure S2**: **Number of eggs per clutch, larval performance, and survival of *Ostrinia nubilalis* on control and EPN-exposed plants**. (A). Number of eggs per clutch on control and EPN-exposed plants or nets when females were allowed to land on the plants (“Free”) or prevented from landing (“Cover”) (n=10 and 21 cages respectively). (B). Larval performance on control and EPN-exposed plants over 7 days (n=13 per treatment). (C). Survival of *Ostrinia nubilalis* larvae on control and EPN-exposed plants after one week of feeding (n=13 per treatment). ns: non-significant.

# Supplementary Figure S3.

**Supplementary Figure S3**: **Transcriptomic response of maize plants to EPN-exposure.** (A). Venn diagram illustrating the number of differentially expressed genes (DEGs) in control and EPN-exposed plants in transcriptomic analyses (n = 5). (B). Kyoto Encyclopaedia of Genes and Genomes (KEGG) pathway enrichment analysis, where the size and colour of the bubbles represent the gene number and Q-value, respectively. (C). Gene Ontology (GO) term enrichment analyses of molecular function (MF), biological processes (BP), and cellular components (CC), respectively. Some of the most relevant terms in the GO are highlighted. Network analyses of (D) down- and (E) upregulated genes, respectively. EPN exposure was achieved by adding 2’000 *Heterorhabditis bacteriophora* nematodes for 72 h in the soil (n = 5 per treatment). NEDD8: Neural precursor cell expressed, developmentally down-regulated8. TRAMP complex: Trf4/Air2/Mtr4p Polyadenylation complex. TOG: Tumor Overexpressed Gene. TPL: TOPLESS. Jas TPL-binding domain: TPL-binding domain in jasmonate signalling. Mei2: Meiotic 2. YT521-B: YT521-B Homolog. XendoU: *Xenopus* Endoribonuclease U. KPTA: Kinase-Phosphotransferase A. TPT1: tRNA Phosphotransferase 1. snoaL: Small Nucleolar RNA-Like. CND41: Chloroplast Nucleoids DNA-binding protein 41. Fes1: Functionally Essential for Stress response 1. AIG2: AvrRpt2-Induced Gene 2. WTAP: Wilms Tumor 1-Associating Protein. MUM2: Methyltransferase-like protein 2. DAZAP1: Deleted in Azoospermia-Associated Protein 1. HSP70: Heat Shock Protein 70. HSP90: Heat Shock Protein 90. SWAP: Suppressor of White Apricot. SURP: Serine/Arginine-Rich Protein. rbbp8: Retinoblastoma Binding Protein 8. VASt: Vps27, Hrs, and STAM domain.

# Supplementary Figure S4.

**Supplementary Figure S4: Metabolomic response of maize plants to EPN-exposure**. (A) Principal component analysis (PCA) of the volatile profile of maize plants (n = 5) (B) PCA of the metabolomic profile of maize leaves (n = 13). (C) Levels of phytohormones present in maize leaves (n = 13). (D) Concentrations of free essential amino acids present in maize leaves (n = 13). (E) Concentrations of soluble sugars present in maize leaves (n = 13). Bars indicate mean +/- standard error. ns: not significant. Stars indicate significant differences (*= *p*-value < 0.05, ns: not significant). FW: Fresh weight.
